# Interferon-alpha Induces High Expression of APOBEC3G and STAT-1 *in Vitro* and *in Vivo*

**DOI:** 10.3390/ijms11093501

**Published:** 2010-09-20

**Authors:** Hui Chen, Lu-Wen Wang, Yan-Qing Huang, Zuo-Jiong Gong

**Affiliations:** Department of Infectious Diseases, Renmin Hospital of Wuhan University, Wuhan 430060, China; E-Mails: chenh8253@163.com (H.C.); wangluw8253@163.com (L.W.); huangyq@163.com (Y.H.)

**Keywords:** STAT-1, interferon-alpha, APOBEC3G, HepG2.2.15 cell, chronic hepatitis B

## Abstract

To investigate whether the JAK-STAT (Janus kinase-signal transducers and activators of transcription) pathway participates in the regulation of APOBEC3G (Apolipoprotein B mRNA-editing enzyme, catalytic polypeptide-like 3G) gene transcription and to study the molecular mechanisms of interferon resistance in patients with chronic hepatitis B (CHB), changes in APOBEC3G and STAT-1 expression levels in HepG2.2.15 cells after treatment with various concentrations of IFN-α, were detected using real-time RT-PCR and Western-blot. In addition, the differences in STAT-1 and APOBEC3G expression in liver tissues were also observed in patients with different anti-viral responses to IFN-α. It is found that IFN-α suppressed HBV replication and expression markedly in HepG2.2.15 cells, and simultaneously enhanced APOBEC3G expression in a dose- or time-dependent manner within a certain range. Moreover, a corresponding gradual increase in STAT-1 expression levels was also observed. The expression levels of STAT-1 and APOBEC3G in the liver of CHB patients with a complete response to IFN-α are significantly higher than that of the patients with non-response to IFN-α treatment. It is suggested that inducing intracellular APOBEC3G expression may be one of anti-HBV mechanisms of IFN-α, and IFN-α-induced APOBEC3G expression may be via the JAK-STAT signaling pathway. Moreover, interferon resistance may be related to the down-regulation of STAT-1 expression in the patients who had non-response to IFN-α treatment.

## 1. Introduction

Apolipoprotein B mRNA-editing enzyme catalytic polypeptide (APOBEC) family members consist of a series of cytidine deaminases, including APOBEC1, -2, -3A, -3B, -3C, -3E, -3F, -3G and activation-induced cytidine deaminase (AID). APOBEC3G, a protein member of the APOBEC superfamily, has been suggested to play an important role in innate anti-viral immunity. APOBEC3G is expressed markedly in liver [1]. APOBEC3G can induce nucleoside mutations from deoxycytidine to deoxyuridine in the viral genome [2]. APOBEC3G has been identified as an intrinsic factor able to restrict replication of human immunodeficiency virus type 1 (HIV-1) lacking the viral accessory protein Vif [3,4]. Recently, it has been revealed that APOBEC3G also inhibits hepatitis B virus (HBV) replication in cultured human hepatocytes [5–8]. However, the expression level of APOBEC3G is normally low in the human liver [9]. Thus, the role of APOBEC3G protein in host cell defenses against HBV infection in human hepatocytes remains unclear.

Interferon-alpha (IFN-α) is a main cytokine induced in the innate immune response directed against viral infection [10]. IFN-α is rapidly induced with a high expression level and secreted into the blood circulation in response to viral infection in many types of cells, and then binds to a specific cell surface receptor and triggers intracellular reactions that lead to the transcriptional induction of IFN-stimulated genes (ISGs) [11]. The expression products of ISGs are many kinds of antiviral proteins including dsRNA-activated protein kinase (PKR), GBP-2, MxA, RANTES, and 2′-5′ oligoadenylate synthetase (OAS) [12], which are able to inhibit viral replication at the levels of penetration, uncoating, mRNA synthesis, protein synthesis, or assembly [13]. Currently, IFN-α is widely used for the treatment of chronic HBV infection [14]. However, the molecular mechanisms of IFN-α suppression of HBV replication in hepatocytes remain poorly understood.

IFN-mediated signal transduction and transcriptional activation is performed mainly through the Janus kinase-signal transducers and activators of transcription (JAK-STAT) signaling pathway [15]. It has been shown that STAT type 1 (STAT-1) is a critical molecule that plays a role of molecular messenger in the JAK-STAT signaling pathway, any regional damages of which may reduce the antiviral efficacy of IFN-α [16].

In the present study, we observed changes in APOBEC3G and STAT-1 expression levels in HepG2.2.15 cells after treatment with various concentrations of IFN-α. With different responses to antiviral therapy with IFN-α, the differences of STAT-1 expression in the liver tissues of patients with chronic hepatitis B were observed, in order to investigate whether the JAK-STAT signal pathway participates in the regulation of APOBEC3G gene transcription and to study the molecular mechanisms of IFN resistance in patients with chronic hepatitis B.

## 2. Materials and Methods

### 2.1. Cell Culture and IFN-α Stimulation

The HepG2.2.15 cell line was transfected with a plasmid containing two head-to-tail dimers of the hepatitis B virus (HBV) genome, which can secrete HBV DNA, HBsAg and HBeAg into medium stably and continuously in the course of cell culture [17]. HepG2.2.15 cells were conventionally cultured in Dulbecco’s modified Eagle’s medium (DMEM, GIBCO/BRL Co., USA) supplemented with 10% fetal bovine serum (FBS, GIBCO/BRL Co., USA), 4 mmol/L *L*-glutamine, 100 U/mL penicillin and 100 μg/mL streptomycin under a humidified atmosphere of 5% CO_2_ at 37 °C. HepG2.2.15 cells were seeded into 12-well plates at a density of 5 × 10^5^ cells per well in DMEM supplemented with 10% FBS, and then were treated with various concentrations of IFN-α (0 U/mL, 1 U/mL, 10^1^ U/mL, 10^2^ U/mL, 10^3^ U/mL, 10^4^ U/mL) for 8 hours, or with IFN-α of 10^3^ U/mL for 2, 4, 6, 8, 10, 12 hours. The cells and culture supernatants were collected for subsequent experiments at each time point. Every five-culture well was set for each treatment.

### 2.2. Detection of HBsAg, HBeAg and HBV DNA in Culture Medium

After HepG2.2.15 cells were treated with 10^4^ U/mL IFN-α for 8 hours, HBsAg and HBeAg in the culture medium were measured quantitatively by electrochemiluminescence immunoassay (ECLIA) (Elecsys 2010, Roche Diagnostics Shanghai Ltd.). HBV DNA in culture medium was also detected by real-time fluorescent quantitative PCR (Lightcycler, Roche Diagnostics Shanghai Ltd.).

### 2.3. Patients and Liver Tissue Specimens

Thirteen patients with chronic hepatitis B (CHB) were selected from the clinic or hospitalization unit at the department of infectious diseases, Renmin Hospital of Wuhan University during February 2008 and October 2009. All patients enrolled fulfilled the following criteria before antiviral therapy with recombinant human IFN-α-2b: alanine aminotransferase (ALT) ranged from 80 to 400 U/L, normal total bilirubin (TBil), HBV DNA ranged from 10^5^ to 10^7^ copies/mL, and positive HBsAg and HBeAg. All patients were treated with recombinant human IFN-α-2b (trade name: Andafen, Anhui Anke Biotechnology (Group) Co., Ltd, China), which was injected intramuscularly at a dose of 5 MU three times a week for 24 weeks. Among the total patients, 5 cases had complete response to IFN-α therapy (as group A) and 8 patients had non-response to IFN-α treatment (group B). The criteria of a complete response to IFN-treatment were HBV DNA loads <5 × 10^2^ copies/mL, HBeAg serum conversion, and normal ALT. The criteria of non-response to IFN-treatment were HBV DNA loads decrease ≤3 1og_10_ or increase without HBeAg serum conversion, and ALT > 40 U/L. There were no statistical differences in HBV DNA loads and ALT level between the patients of the two groups before treatment. All patients were genotype C. The patients of both groups were all male, aged 18–42 years with a median age of 26 years. Liver tissue samples were obtained from the patients via percutaneous Menghini needle extraction after antiviral treatment with IFN-α. Small portions of the livers were kept frozen at −80 °C for reverse transcriptase-polymerase chain reaction (RT-PCR) or Western blot. Other portions were separated and immersed in 10% buffered formalin solution for histological examination. Informed consent of the patients was obtained in accordance with institutional guidelines and the local ethics committee.

### 2.4. Histopathological Examination

Liver specimens were fixed in 10% formaldehyde for 12–24 hours, embedded in paraffin, sliced into sections of 5 μm thickness and stained with hematoxylin and eosin (H&E) to evaluate the pathological changes of liver tissue under light microscope.

### 2.5. Real-Time RT-PCR

RNA was isolated from the HepG2.2.15 cells collected at each time point by using TRIzol reagent (Invitrogen Co., USA). Residual DNA was removed from the RNA sample by using the DNAFREE RNA Kit (Zymo Research Corporation, USA). The amount of RNA was quantified by using a RiboGreen^®^ RNA Quantitation Kit (Molecular Probes, Inc., USA). Two microgram of total cellular RNA was reverse-transcribed into single-stranded complementary DNA (cDNA) by using a random primer hexamer (Promega Corporation, USA) and a MultiScribe™ Reverse Transcriptase (Applied Biosystems, USA) in a two-step RT-PCR reaction. The APOBEC3G, STAT-1 and HBV mRNA levels were determined in a real-time PCR with the SYBR Green I (Applied Biosystems, USA) by using LightCycler (Roche Diagnostics Shanghai Ltd.). GAPDH was used as a reference housekeeping gene and an internal control. Primers were designed using the Primer Express program (PerkinElmer Inc., USA) according to the nucleotide sequence from GenBank. The primers for APOBEC3G (284 bp) were forward 5′-GCT GTG CTT CCT GGA CGT GA-3′ and reverse 5′-GGT GGT CCA CAA AGG TGT CCC-3′; for STAT-1 (200 bp) were forward 5′-CGA AGA GCG ACC AAA AAC AG-3′ and reverse 5′-TGC TGG AAG AGG AGG AAG GT-3′; for HBV (294 bp) were forward 5′-CTT CAT CCT GCT GCT ATG-3′ and reverse 5′-CAC TGA ACA AAT GGC AC-3′; for GAPDH (238 bp) were forward 5′-TTC ACC ACC ATG GAG AAG GC-3′ and reverse 5′-GGC ATG GAC TGT GGT CAT GAG-3′. Thermal cycling conditions included pre-incubation at 50 °C for 2 min, 95 °C for 10 min followed by 35 cycles of 94 °C for 20 s, 54 °C for 30 sec, 72 °C for 30 s. LightCycler collected data automatically and analyzed the value of Threshold Cycle (Ct). The fold changes of APOBEC3G, STAT-1 and HBV mRNA expression were detected by using 2^−ΔΔCt^ method [18].

The mRNA levels of STAT-1 and APOBEC3G in the patients who had different responses to IFN-treatment were also determined by real-time RT-PCR with the same methods as mentioned above from approximate 100 mg of frozen liver tissue specimens.

### 2.6. Western Blot Assay

HepG2.2.15 cells collected at each time point were lysed in cold RIPA buffer (Pierce Biotechnology, Inc., USA), supplemented with Halt™ Protease Inhibitor Cocktail (Pierce Biotechnology, Inc., USA). Whole cell lysates were obtained by subsequent centrifugation at 15,000 × *g* for 10 min at 4 °C. Protein concentrations were determined by using Bradford Protein Assay Kit with bovine serum albumin (BSA) as standard (SinoBio Biotech Co., Ltd. Shanghai, China). 50 μg of protein extracts were subjected to 12% SDS-PAGE and then transferred to a Protran^®^ nitrocellulose membrane (Schleicher & Schuell BioScience GmbH, Whatman Group, Germany). The membrane was incubated with primary antibody (rabbit anti-human APOBEC3G polyclonal antibody, Aviva Systems Biology Co., USA; or rat anti-human STAT-1 monoclonal antibody, Santa Cruz Co., USA) at 4 °C overnight after blocking with a 10% BSA solution. The membrane was washed with tris-buffered saline Tween (TBST) buffer and then incubated with a secondary antibody (goat anti-rabbit or goat anti-rat horseradish peroxidase (HRP)-conjugated IgG, Zhongshan Golden Bridge Biotechnology Co., Ltd., Beijing, China) for 2 hrs at room temperature, and finally detected by chemiluminescence using Enhanced NuGlo™ Chemiluminescent Substrate Kit (Alpha Diagnostic Intl. Inc., USA) followed by autoradiography and densitometry analysis. Membranes were also probed for β-actin as additional loading control.

One hundred milligram of liver tissue samples from the patients who had different responses to IFN-treatment were crushed in a liquid nitrogen-cooled grinding bowl, and protein levels of STAT-1 and APOBEC3G in liver were tested by Western blot using the same methods as mentioned above.

### 2.7. Statistical Analysis

All data were presented as means ± SD. Differences among groups were assessed by using unpaired Student *t* test and one-way ANOVA. *P* value less than 0.05 was considered to be statistically significant. Calculations were performed with the SPSS version 13.0 statistical software package (SPSS, Chicago, USA).

## 3. Results

### 3.1. Changes in APOBEC3G Expression in HepG2.2.15 Cells

**Figure 1:**
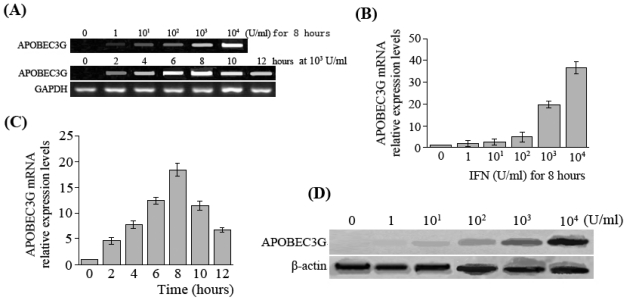
APOBEC3G expression in HepG2.2.15 cells in response to IFN-α stimulation. (**A**) RT-PCR electrophotogram of APOBEC3G mRNA after HepG2.2.15 cells were treated with various concentrations and times of IFN-α; (**B**) Detection of APOBEC3G mRNA by real-time fluorescent quantitative RT-PCR after HepG2.2.15 cells were treated with various concentrations of IFN-α (0 U/mL, 1 U/mL, 10^1^ U/mL, 10^2^ U/mL, 10^3^ U/mL, 10^4^ U/mL) for 8 hours; (**C**) Detection of APOBEC3G mRNA by real-time fluorescent quantitative RT-PCR after HepG2.2.15 cells were treated with IFN-α of 10^3^ U/mL for 2, 4, 6, 8, 10, 12 hours; (**D**) Detection of APOBEC3G protein by Western-blot after HepG2.2.15 cells were treated with various concentrations of IFN-α for 8 hours.

RT-PCR quantitation results revealed that the APOBEC3G mRNA levels were very low in HepG2.2.15 cells without IFN-α treatment. However, APOBEC3G mRNA levels significantly increased (36-fold) in HepG2.2.15 cells with 10^4^ U/mL IFN-α treatment. When the concentration of IFN-α was increased, APOBEC3G mRNA expression also gradually increased. When concentration of IFN-α was 10^4^ U/mL, the APOBEC3G mRNA level in HepG2.2.15 cells was the highest, which had a statistical difference compared with HepG2.2.15 cells treated with 10^3^ U/mL (36.658 ± 2.625 *vs*. 19.565 ± 1.564, *t* = 12.509, *P* < 0.01) (Figure 1A, B). With the prolongation of IFN-α stimulation time, APOBEC3G mRNA level in HepG2.2.15 cells rose, reaching a peak at 8 hours, which had a statistical difference compared with 6 hours (18.423 ± 1.205 *vs*. 12.356 ± 0.695, *t* = 9.752, *P* < 0.01). Subsequently, APOBEC3G mRNA level decreased gradually, and there was no statistical difference in APOBEC3G mRNA level between 10 hours and 6 hours (11.375 ± 0.878 *vs*. 12.356 ± 0.695, *t* = 1.959, *P* > 0.05) (Figure 1A, C). The results of Western blot assays further showed a dose-dependent effect of IFN-α on APOBEC3G protein expression (Figure 1D).

### 3.2. Changes in STAT-1 Expression in HepG2.2.15 Cells

**Figure 2:**
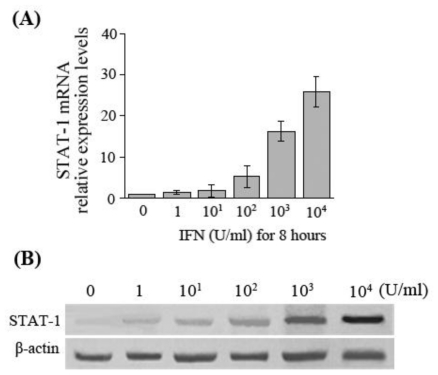
STAT-1 expression in HepG2.2.15 cells after IFN-α stimulation. (**A**) Detection of STAT-1 mRNA by real-time fluorescent quantitative RT-PCR after HepG2.2.15 cells were treated with various concentrations of IFN-α; (**B**) Detection of STAT-1 protein by Western-blot after HepG2.2.15 cells were treated with various concentrations of IFN-α for 8 hours.

The results of both real-time RT-PCR and Western blot assays showed that the expression levels of STAT-1 mRNA and protein increased with an increasing stimulus concentration of IFN-α in HepG2.2.15 cells, which was consistent with the change trends of the dose-dependent increase in APOBEC3G expression (Figure 2).

### 3.3. Changes in HBV Replication and Expression in HepG2.2.15 Cells

When HepG2.2.15 cells were treated with 10^4^ U/mL IFN-α for 8 hours, the levels of HBsAg, HBeAg and HBV DNA in culture medium, and HBV mRNA in cells were all significantly lower than that of HepG2.2.15 cells without IFN-α stimulation (Table 1). This suggests that IFN-α suppressed HBV replication and expression significantly in HepG2.2.15 cells.

### 3.4. Histopathological Changes in the Liver of CHB Patients Wth Different Responses to IFN-α

**Figure 3:**
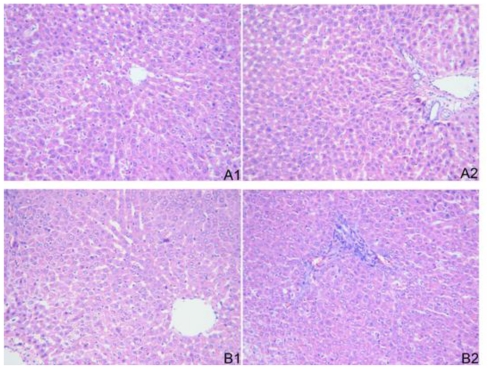
Representative light microscopy images of liver tissue stained with from the patients who had complete response or no response to IFN-α antiviral therapy (magnification ×200). **A1** and **A2**: Liver tissue of patients who had a complete response to antiviral therapy by IFN-α displayed a near normal hepatic lobule structure, arrayed radiatively around the central vein, the Disse cavity was clear and well-distributed, and there was no obvious periportal inflammatory cell infiltration. **B1** and **B2**: Liver tissue of patients who had non-response to antiviral therapy by IFN-α showed hepatocytes with degeneration in hepatic lobule and periportal inflammatory cell infiltration.

The liver tissue of the patients in group A (complete response to IFN-α) had a near normal hepatic lobule structure, which showed that hepatocytes were not degenerated or necrotic, and arrayed radiatively around the central vein, the Disse cavity was clear and well-distributed, and there was no obvious periportal inflammatory cell infiltration. The liver tissue of the patients in group B (non-response to IFN-α) showed hepatocytes with degeneration in the hepatic lobule, and periportal inflammatory cell infiltration was obvious (Figure 3).

### 3.5. STAT-1 and APOBEC3G Expression in the Liver Tissue of CHB Patients Who Had Different Responses to IFN-α

**Figure 4:**
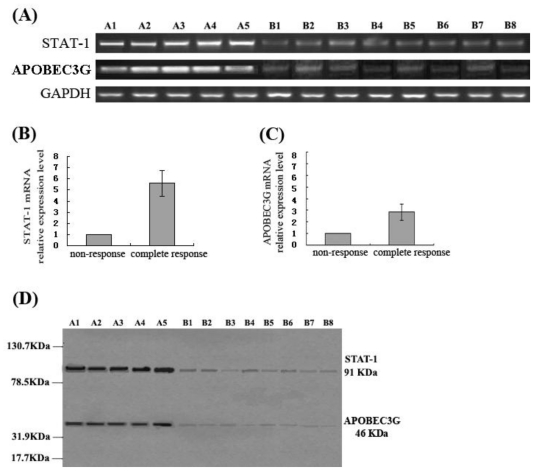
Expression of STAT-1 and APOBEC3G in liver tissue of the patients who had complete response, or no response to antiviral therapy by IFN-α after treatment. (**A**) RT-PCR electrophotogram of STAT-1 mRNA and APOBE3G mRNA. (**B**) Detection of STAT-1 mRNA by real-time fluorescent quantitative RT-PCR. (**C**) Detection of APOBEC3G mRNA by real-time fluorescent quantitative RT-PCR. (**D**) Detection of STAT-1 protein and APOBEC3G protein by Western blot. A1-A5: Five patients who had a complete response to IFN-α; B1–B8: Eight patients who had non-response to IFN-α.

The results of both RT-PCR and Western blot showed that STAT-1 and APOBEC3G expression levels in the liver tissue of the patients in group A were significantly higher than that of the patients in group B, which revealed that the CHB patients with a complete response to antiviral therapy by IFN-α had a higher expression of STAT-1 and APOBEC3G in the liver, while the expression levels of STAT-1 and APOBEC3G in the liver of the patients without response to IFN-α were significantly lower (Figure 4).

## 4. Discussion

In this study, the results showed that expression of APOBEC3G was very low in HepG2.2.15 cells, but IFN-α was able to induce HepG2.2.15 cells to express APOBEC3G at a high level. Moreover, APOBEC3G expression levels increased is a dose- or time-dependent manner within a certain range in HepG2.2.15 cells after the treatment with IFN-α. These results agree with the findings of Tanaka [19] and Bonvin [20]. It is suggested that under normal circumstances, the liver cells may not express APOBEC3G, or the expression is relatively low, but cytokines such as IFN-α can induce production of APOBEC3G during the course of HBV infection.

The results of this study demonstrated that IFN-α suppressed HBV replication and expression markedly in HepG2.2.15 cells, and simultaneously enhanced APOBEC3G expression. Moreover, this study also showed that the CHB patients with complete response to antiviral therapy with IFN-α had a higher APOBEC3G expression in the liver, while the levels of APOBEC3G expression in the liver of the patients without response to IFN-α treatment were significantly lower. Previous studies have revealed that APOBEC3G also plays a role in inhibiting HBV replication [5–8], so the data of our study suggest that the anti-HBV effects of IFN-α could be performed by enhancing intracellular APOBEC3G expression, which probably is one of the antiviral mechanisms of IFN-α.

However, recent studies demonstrated that in spite of the induction of APOBEC3G in hepatoma cells exposed to IFNs, the cytokine-mediated inhibition of HBV replication is not affected when the expression of APOBEC3G is suppressed by combining RNA interference and the Vif protein of HIV-1 [21]. The studies further demonstrated that in HBV-transgenic murine APOBEC3 (muA3) knockout mice, IFN induction blocked HBV DNA production as efficiently as in control HBV-transgenic muA3-competent animals [22]. From these data, it is concluded that IFN does not exert its antiviral effect through induction of APOBEC3, which suggest that APOBEC3 is not an essential mediator of the IFN-mediated inhibition of HBV *in vivo*. The downstream effectors of the anti-HBV effect of IFN thus remain to be identified in further studies.

While demonstrating that APOBEC3G is not required for the inhibition of HBV by IFN-α both in cell culture and in transgenic mice, these results do not imply that antiviral APOBEC3G have no relevance for HBV biology and therapy. This study showed that the APOBEC3G protein is weakly expressed in HepG2.2.15 cells, but its expression levels increased in a dose- or time-dependent manner in IFN-treated HepG2.2.15 cells. So APOBEC3G may have a greater impact on HBV replication upon stronger expression, which may occur in the infected liver. Thus, it is not excluded that APOBEC3G acts in concert with other yet-undefined IFN-induced effectors during the clearance of HBV *in vivo*.

To assess further the role of APOBEC3G in the IFN-mediated clearance of HBV, it might be revealing to compare levels of APOBEC3G in the livers of uninfected, acutely infected, and chronically infected individuals. Informative data might also be obtained by looking at possible interindividual differences in the sequences of APOBEC3G promoters, particularly with respect to IFN-responsive elements. As well, an evaluation of the sensitivity of HBV strains isolated at various stages of the disease to APOBEC3G could be indicative.

The rate of complete response to IFN-α treatment is only unsatisfied 30~33% in the patients with chronic hepatitis B (CHB) [23], which is suggested to be related to interferon resistance existing in the patients’ bodies. The exact mechanisms of the interferon resistance remains unclear, however, at least three factors are involved including host, virus and interferon. It has been suggested that the host factor is probably related with down-regulation of IFN receptors or IFN signal transduction [24]. The JAK-STAT (Janus kinase-signal transducers and activators of transcription) system is a major signaling pathway of alpha interferon-mediated signal transduction and transcriptional activation. Among the various STAT proteins, STAT-1 is a key signaling component of IFN-α response [25]. In this pathway, following engagement of the heterodimeric receptor at the cell surface with IFN-α, JAKs associated with IFN-α receptors phosphorylate both STAT-1 and STAT-2. As a result, an IFN-stimulated gene factor 3 (ISGF3) complex forms, which contains STAT-1, STAT-2 and a third transcription factor called IRF9 (also known as p48). ISGF3 is translocated from the cytosol into the cell nucleus, in which it binds to specific nucleotide sequences called IFN-stimulated response elements (ISREs) in the promoters of target genes to induce transcription of those IFN-stimulated genes (ISGs) [26,27].

In the present study, the results manifested that at the same time of IFN-α-induced upregulation of APOBEC3G expression in HepG2.2.15 cells, a corresponding gradual increase of STAT-1 mRNA and protein expression levels was also observed. Whether it is suggested that IFN-α-induced APOBEC3G expression is via JAK-STAT signaling pathway is not yet fully established by our experiments. The relationship between them and the exact mechanism remain to be further studied.

In addition, the differences of STAT-1 expression in liver tissue of patients with chronic hepatitis B who had different responses to antiviral therapy with IFN-α were also investigated in this study. The results showed that the expression levels of STAT-1 in the liver of the CHB patients with a complete response to IFN-α are significantly higher than that of the patients with non-response to IFN-α. It is suggested that interferon resistance was related with the down-regulation of STAT-1 expression in the patients who had non-response to IFN-α. What causes low expression of STAT-1 in the patients with non-response to IFN-α is unclear. We hypothesize the reasons might be STAT-1 deficiency or/and effects of HBV on STAT-1 in those patients, which remain to be further studied to identify the exact mechanism of the JAK-STAT signaling pathway in anti-HBV effects of IFN-α. In conclusion, clarifying the mechanisms of IFN resistance could provide a new strategy for enhancing the anti-HBV effects of IFN-α.

## 5. Conclusions

We report that IFN-α suppressed HBV replication and expression markedly in HepG2.2.15 cells, and simultaneously enhanced APOBEC3G expression in a dose- or time-dependent manner within a certain range. Moreover, a corresponding gradual increase of STAT-1 expression levels was also observed. The expression levels of STAT-1 and APOBEC3G in the liver of the CHB patients with a complete response to IFN-α are significantly higher than those of the patients with non-response to IFN-α treatment. The study results suggest that inducing intracellular APOBEC3G expression may be one of anti-HBV mechanisms of IFN-α, and IFN-α-induced APOBEC3G expression may be via the JAK-STAT signaling pathway. Moreover, interferon resistance may be related with the down-regulation of STAT-1 expression in the patients who had non-response to IFN-α treatment.

## Figures and Tables

**Table 1:** Changes in the levels of HBV replication and expression in HepG2.2.15 cells (mean ± SD, *n* = 5).

HepG2.2.15 cells	HBsAg (P/N values)	HBeAg (P/N values)	HBV DNA (lg copies/mL)	HBV mRNA (2^−ΔΔct^ values)
0 U/mL IFN-α for 8 h	5.92 ± 0.05	10.44 ± 0.16	6.32 ± 0.15	1.00
10^4^ U/mL IFN-α for 8 h	2.37 ± 0.04	5.41 ± 0.08	3.45 ± 0.28	0.41 ± 0.13
